# Study of Flow and Zinc Dross Removal in Hot-Dip Galvanizing with Combined Traveling Magnetic Field

**DOI:** 10.3390/ma17194799

**Published:** 2024-09-29

**Authors:** Xianwen Luo, Haibiao Lu, Yunbo Zhong, Weili Ren, Zuosheng Lei

**Affiliations:** State Key Laboratory of Advanced Special Steel & Shanghai Key Laboratory of Advanced Ferrometallurgy, Shanghai University, Shanghai 200444, China; luoxw@shu.edu.cn (X.L.); yunboz@staff.shu.edu.cn (Y.Z.); wlren@staff.shu.edu.cn (W.R.)

**Keywords:** hot-dip galvanizing, zinc dross, electromagnetic-driven flow, air-knife jet, flow field

## Abstract

The removal of zinc dross, which continuously generates and partially floats on a molten zinc surface, has been a persistent challenge during hot-dip galvanizing. Herein, a three-dimensional mathematical model coupled with the electromagnetic field, flow field and air-knife jet flow was established to investigate the flow and zinc dross removal in a zinc pot. Two types of traveling magnetic field combined modes (Mode 1 and Mode 2) were compared. The surface dross removal efficiency was introduced to evaluate the ability of the zinc flow field to compel the movement of zinc dross. The research findings indicate that, in comparison to the influence of strip steel line speed, both the electromagnetic field and air-knife jet have a more pronounced effect on altering the flow characteristics of a molten zinc at surface. The dross removal efficiency for Mode 1 is much far superior to that of Mode 2. With an increase in the driving current, the dross removal efficiency increases while the excessive driving current cannot promote the dross removal efficiency significantly.

## 1. Introduction

Continuous hot-dip galvanizing of strip steel is one of the most widespread, economical and effective technologies used to protect steel from external corrosion by means of the principle of cathodic protection [1]. In the hot-dip galvanizing process, the reductively annealed strip enters a zinc bath at a temperature range of 450~480 °C with the synergistic operation of the roller apparatus completing the galvanizing on the strip surface [2]. The zinc coating thickness is controlled by the air-knife jet above the zinc pot. As the worldwide demand for galvanized steel products continues to grow, the production of lower cost, higher quality hot-dip galvanized steel is facing enormous difficulties and challenges. Generally, zinc dross is a major obstruction to the production of high-quality coatings.

During the hot-dip galvanization process, a specific quantity of aluminum (Al) is incorporated into the zinc pot, which can not only enhance the fluidity of the molten zinc but also endow the substrate material with enhanced welding and processing characteristics [3]. However, this process will inevitably form the zinc dross as an Al–Fe–Zn ternary compound [4,5]. According to their distribution in the zinc bath, the zinc dross can be divided into top dross (η-Fe_2_Al_5_Zn_x_) with a density of about 4200 kg/m^3^, suspended dross with a density close to 6700 kg/m^3^, and bottom dross (ζ-FeZn_13_, δ-FeZn_10_Al_x_) with a density higher than liquid Zn [6,7]. These intermetallic phases can transform dynamically based on the variation of the concentration of Al [8]. During this process, some of the zinc dross would be entrapped by the steel strip, and finally cause pinpoint defects or linear defects on its surface [9]. Prior investigations have demonstrated a strong correlation between the trajectory of the zinc dross and flow field in the zinc bath [10], while the optimization of the flow field offers the potential to improve the comprehensive distribution of zinc dross, and to mitigate the surface defects of the steel strip. Consequently, it is of great importance to investigate the internal flow field within the zinc pot.

A considerable number of past studies have attempted to reveal the flow characteristics in a zinc bath through physical experiments [11]. Kurobe et al. [12] used a laser doppler velocimetry (LDV) system to analyze the flow pattern inside a zinc pot model and the movement of the dross was visualized. Lee et al. [9] established a water model to measure transient velocity fields in a transparent zinc pot by using particle image velocimetry (PIV), and found that attaching a scraper to the stabilizing roll guides the separated flow from the strip downward, while the uprising flow around the stabilizing roll becomes slow and tranquil. With the development of computational fluid dynamics (CFD), numerical simulations have gradually supplanted physical experiments as the primary method to study the transport phenomena within zinc baths. Kim et al. [13] and Ajersch et al. [14] developed a 3D numerical model to simulate the flow patterns and temperature fields in an industrial galvanizing bath, and evaluated the effect of certain production process parameters on these patterns. Zhou et al. [15] numerically simulated the zinc flow in a galvanizing bath and found that a vortex formed between the snout and the bath side walls increases the risk of dross clinging to the strip surface. However, the aforementioned studies have not accounted for the effects of the air-knife jet process, while this can also influence the flow field, especially near the surface of the zinc bath [16,17,18]. Pfeiler et al. [19] have employed the large-eddy simulation (LES) and volume-of-fluid (VOF) model to simulate the flow structure and impingement dynamics between compressible gas and the zinc coating during the air-knife wiping process, while not considering the interaction between the jet and the zinc bath flow field. Indeed, the air-knife jet serves as a critical operation during the continuous hot-dip galvanizing process of strip steel, which exerts a pivotal influence on the zinc flow and dross track at the strip exit. Therefore, it is necessary to couple the interaction between the air knife and zinc bath.

The molten zinc flow and the formation of zinc dross in hot-dip galvanizing production are interrelated. Even though a considerable number of past studies have been conducted to remove dross effectively, most of them are concentrated on the theoretical analysis and plant in-trial. Generally, the most widely used methods to remove the zinc dross are manual or robotic arm-based approaches. However, both of these approaches exhibit some drawbacks, including increased disturbance of molten zinc levels and suboptimal dross removal performance. It is a widely acknowledged fact that the manipulation of molten metal flow can be effectively regulated by electromagnetic fields [20,21]. Accordingly, the application of an electromagnetic field can similarly facilitate the propulsion of molten zinc, which in turn induces the movement of zinc dross. By applying the non-contact and precise control capabilities of the electromagnetic field, the dross can be promptly and efficiently removed from the vicinity of the strip exit, and transported to either the side walls of the zinc pot or the back of the snout. Xiao et al. [22] have focused on coupling electromagnetic field and flow-field simulations to analyze the flow characteristics of molten zinc within a zinc pot. Nevertheless, this study ignored the effect of the strip and roller apparatus motion as well as the air-knife jet on the flow field. Comprehensive numerical investigations on the zinc flow field under the combined effect of the electromagnetic field and air-knife jet have not been reported.

In this study, a numerical simulation of the flow field in a zinc pot is conducted by incorporating EMDF and considering the influence of the air-knife jet process. A mathematical model is developed to couple the electromagnetic field and the zinc bath flow field. The electromagnetic-driven force and air-knife jet pressure are computed and integrated into the momentum equation as source terms using user-defined functions (UDF). The two modes of EMDF distribution and various driving current parameters are discussed, aiming to proficiently regulate the flow of molten zinc at the surface layer and consequently improving the distribution of zinc dross. Additionally, the surface dross removal efficiency is introduced to evaluate the removal effect of zinc dross.

## 2. Modeling Description

### 2.1. Modeling Assumption

In order to simplify the numerical simulation considering the complexity of the hot-dip galvanizing process, the following assumptions were made:

(1) Given that the frequency of the electromagnetic driving device is 4.5 Hz, the magnetic field is assumed to be quasi-static and the displacement current is not considered [20].
(2) The molten zinc in the zinc pot is treated as an isothermal incompressible Newtonian fluid, and parameters such as the density, viscosity, magnetic permeability, and electrical conductivity of the molten zinc are treated as constants.
(3) The influence of the molten zinc flow on the electromagnetic field is ignored.
(4) The air-knife jet is considered as an incompressible Newtonian fluid.

### 2.2. Governing Equation

A three-dimensional mathematical model was developed to simulate the flow field within a zinc pot in the presence of an electromagnetic field and air-knife jet. The model incorporated the following governing equations for control purposes:

#### 2.2.1. Electromagnetic Model

Maxwell’s equations and Ohm’s law are obtained to solve the magnetic field within the zinc pot, which can be expressed as follows:

(1) $$\nabla \cdot \vec{B}=0$$

(2) $$\nabla \times \vec{E}=-\frac{\partial \vec{B}}{\partial t}$$

(3) $$\nabla \times \vec{H}=\vec{J}$$

(4) $$\vec{J}=\sigma \vec{E}$$

where ∇ is the Hamiltonian operator; $\vec{B}$ is the magnetic flux density, $\vec{E}$ is the electric field strength, $\vec{H}$ is the magnetic field strength, $\vec{J}$ is the induced current density, and *σ* is the electric conductivity.

The Lorentz force induced by the electromagnetic field within the molten zinc is estimated using the time-averaged electromagnetic force, which can be computed utilizing the following equation [23]:

(5) F→EMDF=12ReJ→×B∗→

where ${\vec{F}}_{EMDF}$ is the time-averaged electromagnetic force, $\vec{B^{*}}$ is the complex conjugate of $\vec{B}$, and *Re* is the real part of the complex number.

#### 2.2.2. Fluid Flow Model

The primary control equation for computational fluid dynamics includes the mass conservation equation and momentum conservation equation, which can be derived as follows:

(6) $$\nabla \cdot \left(\rho \vec{u}\right)=0$$

where $\vec{u}$ is the velocity of the fluid, *ρ* is the density of the fluid.

The incompressible Navier–Stokes (N-S) equation is used to describe the momentum conservation:

(7) $$\frac{\partial }{\partial t}\left(\rho \vec{u}\right)+\nabla \cdot \left(\rho \vec{u}\vec{u}\right)=-\nabla p+\nabla \cdot \left(\mu_{eff}\nabla \vec{u}\right)+\rho g+{\vec{F}}_{jet}+{\vec{F}}_{EMDF}$$

where *p* is pressure, *g* is the acceleration of gravity, ${\vec{F}}_{jet}$ is the source term for air-knife jet pressure, and $\mu_{eff}$ is the effective viscosity which can be derived as follows:

(8) $$\mu_{eff}=\mu_{l}+\mu_{t}$$

where $\mu_{l}$ is the molecular viscosity and $\mu_{t}$ is the turbulent viscosity.

### 2.3. Geometry Model and Boundary Conditions

#### 2.3.1. Geometry Model

**Figure 1:**
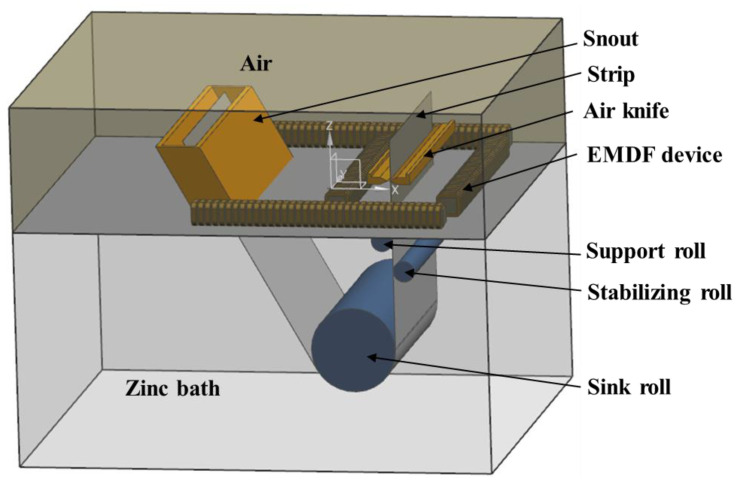
Geometric model of air-knife jet and schematics of zinc pot with electromagnetic-driving device calculation model.

In this study, an industrial zinc pot which consists of a sink roll, stabilizing roll, support roll, strip steel, snout, and an array of air knives was established to investigate the flow pattern within the zinc pot. Four sets of electromagnetic driving devices, composed of an iron core and coil, are laid out above the top of the molten zinc, as shown in Figure 1. Owing to the high grid density near the nozzles in the compressible air jet model, coupling it with the EMDF model to investigate the complex flow field within the zinc pot will inevitably necessitate a considerable investment of computational time and cost. Therefore, the air-knife jet model and EMDF model were decoupled, both utilizing hexahedral structured grids. Grid-independence verification was performed. In order to calculate the air-knife jet behavior and the molten zinc flow pattern more accurately, mesh refinement was applied near the nozzle edges and the roller apparatus, resulting in a total of approximately 2.8 million mesh elements. Table 1 shows the geometric and process parameters for numerical simulation.

#### 2.3.2. Boundary Conditions for the Electromagnetic Field and Flow Field

Electromagnetic simulation: The whole geometry model of the electromagnetic simulation was surrounded by an air cuboid (4.80 m × 4.5 m × 2.3 m) in order to ensure that most of the magnetic flux lines were closed. Magnetically flux parallel boundary conditions were applied on the external surface of the surrounding air cuboid [20].

Flow field simulation: For the zinc bath flow field simulation, the moving wall boundary condition was applied on the strip surface to simulate the movement of strip steel. The upper free surface of the zinc pot was subjected to zero normal velocity and zero shear stress boundary conditions, while the other wall surfaces were assumed to have no-slip boundary conditions [13]. For the air-knife jet simulation, the nozzles were treated as a pressure inlet boundary condition, and the top of the computational domain was set as a pressure outlet boundary condition.

### 2.4. Numerical Solution Procedure

**Figure 2:**
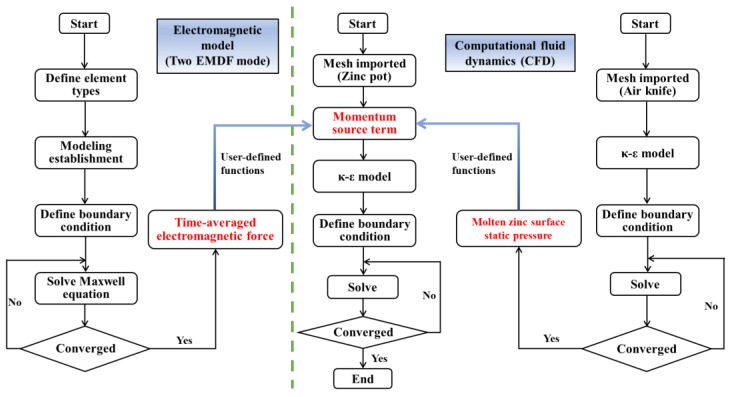
Coupling flow chart of electromagnetic field, flow field, and air knife.

In this study, the sequential coupling steps between the electromagnetic field and flow field are shown in Figure 2. Firstly, the air-knife model was solved to obtain the surface pressure at the zinc pot, and then, Maxwell’s equations were used to solve the electromagnetic fields, current density, and time-averaged electromagnetic forces for two different EMDF modes by using ANSYS Emag 18.2. Finally, the surface pressure and time-averaged electromagnetic force were interpolated into the zinc flow model as a source term of the momentum equation, which was performed on ANSYS FLUENT 18.2 (ANSYS, Inc., Canonsburg, PA, USA). The SIMPLE algorithm was used for the pressure–velocity coupling and the momentum was discretized using the PRESTO! scheme. The other equations were discretized using the first-order upwind scheme. The time-step of every iteration is 0.001, and the convergence criterion was 1 × 10^−5^. The total simulation time was 3 days, which was performed in a workstation with 48 CPU cores at the frequency of 3.6 GHz.

## 3. Results and Discussion

### 3.1. The Validation of the Flow Field and Electromagnetic Field

**Figure 3:**
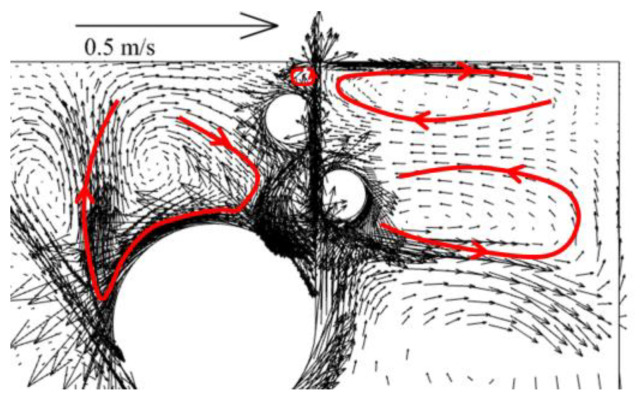
Velocity vectors at the center of the zinc pot in this study.

In order to verify the accuracy of the mathematical model for the flow field, the velocity vector at the center of the zinc pot was compared with that obtained from the Reference [18] with a strip velocity of 1.5 m/s as illustrated in Figure 3. It can be seen that the flow field for the current model is similar to that of the previous study; there are four circulating flow structures located at both sides of the strip steel exit, on the rear side of the stabilizing roll, and above the sink roll.

**Figure 4:**
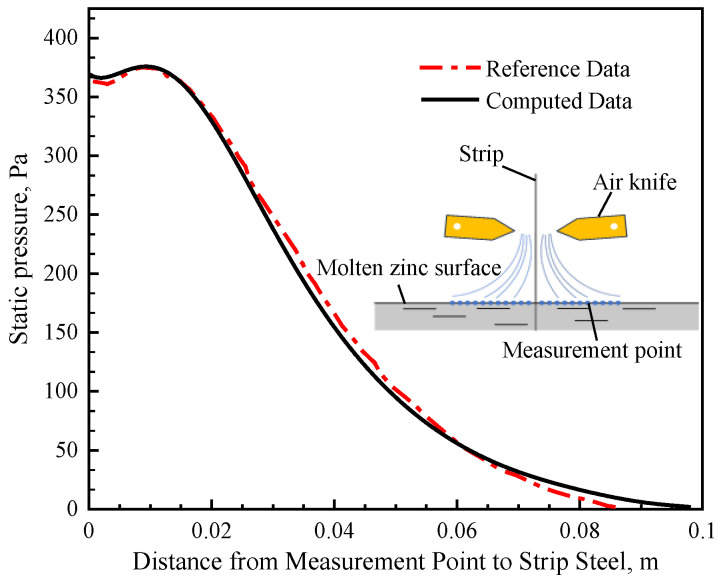
Static pressure distribution of the surface molten zinc with the air knife jet.

The high-pressure jet flow induced by the air knife would affect the flow of molten zinc. To validate the accuracy of the air-knife jet model in this study, the relevant parameters used in the model, such as the knife nozzles to the zinc surface height, the distance of the knife nozzles to the strip, and the steel strip width, were set similarly to the air-knife model reported in Reference [24]. Consequently, specific air-knife operating parameters (20 KPa) were selected, and the calculated relative static pressure distribution on the zinc surface was compared with the measured pressure data from Reference [24], showing a favorable agreement. Two air knives positioned on either side of the strip exit were used to control the coating thickness by directing high-pressure-velocity air jets from the knife nozzles. The jets caused an increase in pressure around the strip exit region, which subsequently affected the zinc flow field [18]. As illustrated in Figure 4, the maximum relative static pressure of 382 Pa was observed at 0.015 m from the strip steel exit. At 0.09 m from the strip steel exit, the relative static pressure tends to approach zero, which further indicates that the jet from air knife can only affect a limited region near the exit of the strip steel.

**Figure 5:**
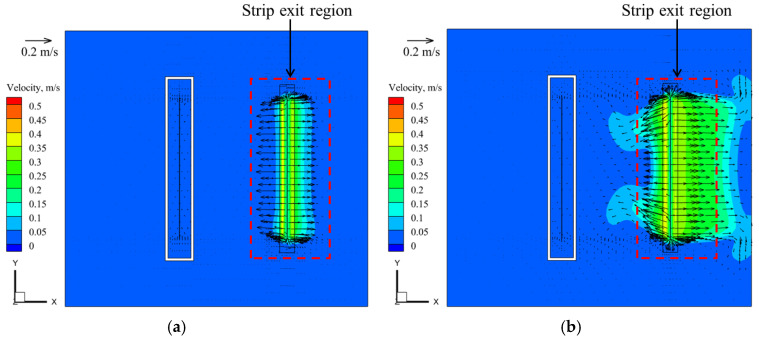
Velocity distribution on the Z = −0.01 m plane (**a**) Without air-knife jet (**b**) 20 KPa air-knife jet pressure.

Figure 5 presents the velocity contour plot at Z = −0.01 m plane of the zinc bath without and with the air-knife jet pressure of 20 KPa, and the strip velocity is 1.5 m/s. It can be seen that, in the absence of the air knife, the weak flow of molten zinc at the strip exit region is caused by the strip motion and stabilizing roll rotation. After the air knife is applied, it is evident that the zinc flow at the strip exit region develops towards both sides of the strip with the influence of jet pressure, making the results more reasonable [17]. Hence, all the subsequent calculations in this study consider the pressure condition of the air-knife jet.

**Figure 6:**
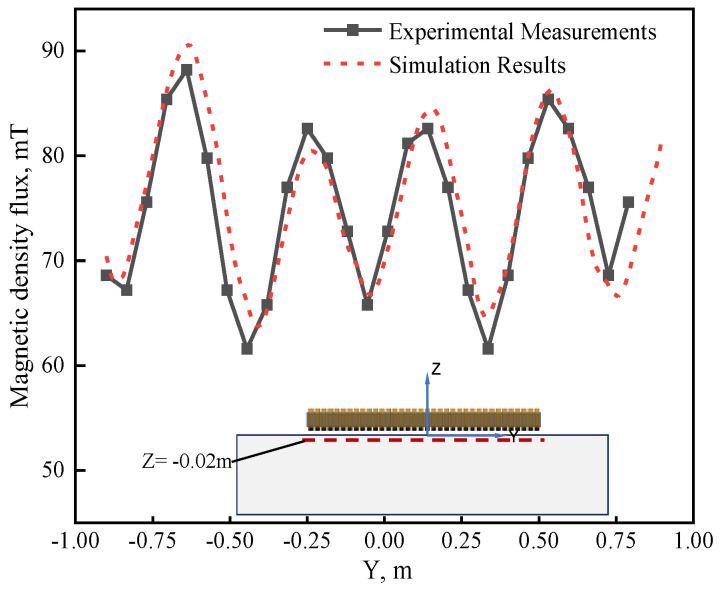
Comparison between the numerical and measured magnetic flux density at a depth of 0.02 m in the zinc bath.

As the magnetic flux density during the galvanizing process with EMDF cannot be measured, modeling without molten zinc is carried out to validate the electromagnetic field. Figure 6 displays the comparison of the magnetic flux density at 20 mm below the surface under the operating condition of 300 A/4.5 Hz, where the measured data were obtained through a CT-3 Tesla meter [25]. A comparison reveals a good agreement between the numerical calculations and the measured data, and the maximum magnetic induction density is approximately 91 mT. Although some differences still exist, this may be attributed to differences between the geometry of the EMDF devices and the constructed geometric model. Additionally, during the actual measurements, temperature fluctuations caused by the devices self-heating resulted in uncertain changes in the material’s magnetic permeability.

### 3.2. Distribution of Electromagnetic Force

**Figure 7:**
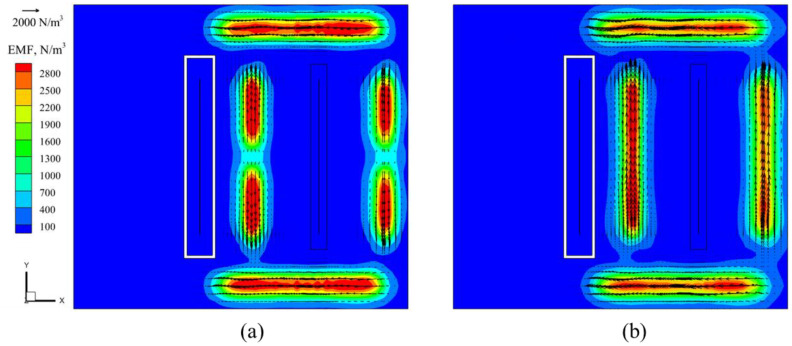
Distribution of electromagnetic force on the zinc bath surface under two different modes: (**a**) Mode 1 (**b**) Mode 2.

Initially, the EMDF devices used in hot-dip galvanizing production operated with a single mode (Mode 2). However, recent studies have introduced a new EMDF mode (Mode 1) [22]. To gain a deeper understanding of the flow capabilities of molten zinc driven by these two modes, in this study, a simulation was conducted on the distribution of electromagnetic force and the penetration depth within molten zinc for both EMDF modes. The distribution of electromagnetic force under two types of EMDF modes at a driving current of 300 A is shown in Figure 7, where it can be seen that the distribution of the electromagnetic force at the upper and lower side of the steel strip is similar, and the main distinction is at the two sides of the strip steel; for Mode 1, the direction of the electromagnetic force is from the center to the edge of the zinc pot, while for Mode 2, the direction is along the positive *Y*-axis, and the maximum electromagnetic force is about 2800 N/m^3^.

**Figure 8:**
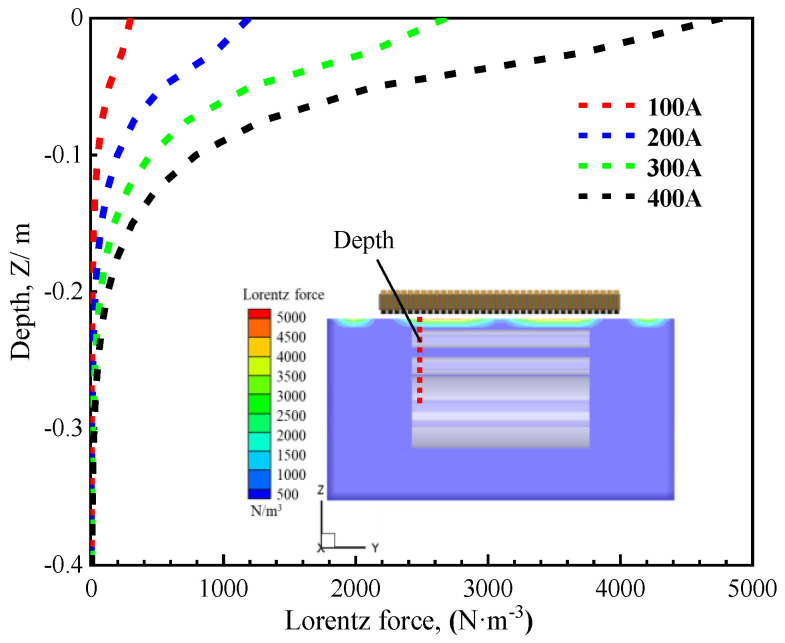
Depth of electromagnetic force in a zinc bath under different driving currents.

Due to the skin effect of the current, the electromagnetic force is concentrated on the surface of the molten zinc, resulting in suboptimal performance of the EMDF. To further investigate the penetration depth of the electromagnetic force, taking Mode 1 as an example, Figure 8 illustrates the distribution of electromagnetic force in the Z-direction and the depth of the effect in a zinc bath under various driving current conditions. It is apparent that the magnitude of the electromagnetic force diminishes rapidly as the depth of the zinc bath increases. Once it descends below 0.1 m from the molten zinc surface, the electromagnetic force becomes considerably weaker. This is primarily associated with the frequency utilized by EMDF devices, simultaneously highlighting that the electromagnetic drive device can exert a significant influence on the molten zinc surface flow.

### 3.3. Effect of EMDF Modes on Zinc Flow Field

**Figure 9:**
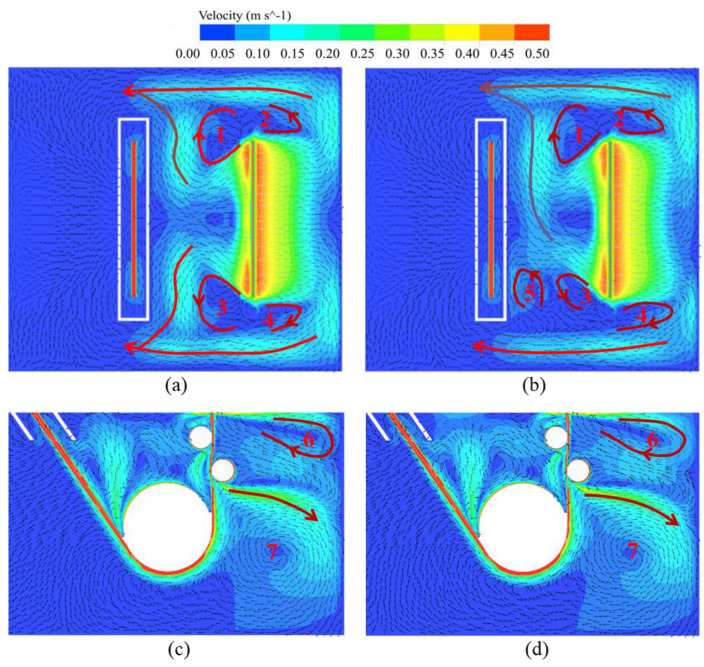
Velocity distribution on plane 1 (Z = −0.01 m) and plane 2 (Y = 0 m) with a driving current of 100 A (**a**,**c**) Mode 1 (**b**,**d**) Mode 2.

Figure 9 illustrates the contour plot of two EMDF modes on the zinc bath flow field, with the computational parameters of an air-knife jet pressure of 20 KPa, and a driving current of 100 A. The pressure from the air-knife jet primarily acts on molten zinc at the front and rear surface near the steel strip exit. When the EMDF is operating, it combines with the air knife, resulting in a more pronounced impact on the flow characteristics of the surface molten zinc. For Mode 1, the surface of the molten zinc is forced to flow directionally towards the side walls of the zinc pot and eventually towards the back of the snout. The overall flow pattern exhibits a symmetrical distribution around the center of the strip (Y = 0), forming four vortex zones (Zone 1–4) near the strip exit, as depicted in Figure 9a. In contrast, for Mode 2, a different flow pattern is observed, with the formation of an additional vortex zone (Zone 5) near the snout region, as shown in Figure 9b. This can be attributed to the lower driving current and unidirectional electromagnetic force along the *Y*-axis, leading to less effective flow control at the end position of the EMDF devices. Furthermore, concerning the internal flow within the zinc bath, both EMDF modes exhibit a typical circulating flow field [14], including the circulating flow 6 in the region before the stabilizing roll and the larger circulating flow 7 near the sinking roll, as illustrated in Figure 9c,d.

**Figure 10:**
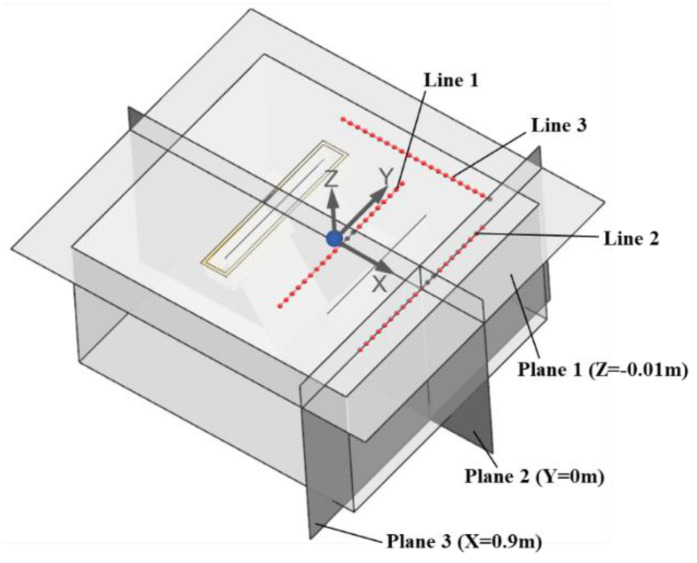
Position of various cross-sections and lines.

**Figure 11:**
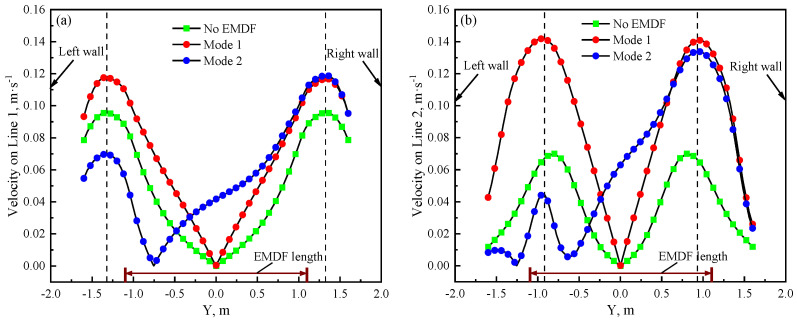
Velocity comparison between cases with and without EMDF (**a**) Line 1 (**b**) Line 2.

To quantitatively analyze the influence of the EMDF modes on the surface molten zinc flow field, Line 1 and Line 2 (as shown in Figure 10), represented by the centerlines of EMDF devices on the front and rear sides of the strip, respectively, are used to investigate the flow velocity in the *Y*-axis direction. Compared to the absence of EMDF, Mode 1 remarkably accelerates the surface molten zinc flow and exhibits a symmetrical distribution, as shown in Figure 11. The velocity exhibits a trend of an initial increase followed by a decrease due to the zinc reflux generated near the zinc pot side walls. However, for Mode 2, in order to overcome the reverse zinc flow caused by the air-knife jet, the flow velocity near the left side wall is even lower than that without EMDF. During the hot-dip galvanizing process, this is an undesirable outcome, as it can lead to an increased likelihood of zinc dross staying or accumulating in that region, posing a higher risk of contact with the strip surface. Moreover, by comparing the maximum velocities under the two modes, it is evident that the maximum velocity position on Line 1 is closer to the zinc pot side walls, whereas on Line 2, it is closer to the strip center.

### 3.4. Effect of Driving Current on the Zinc Flow Field

**Figure 12:**
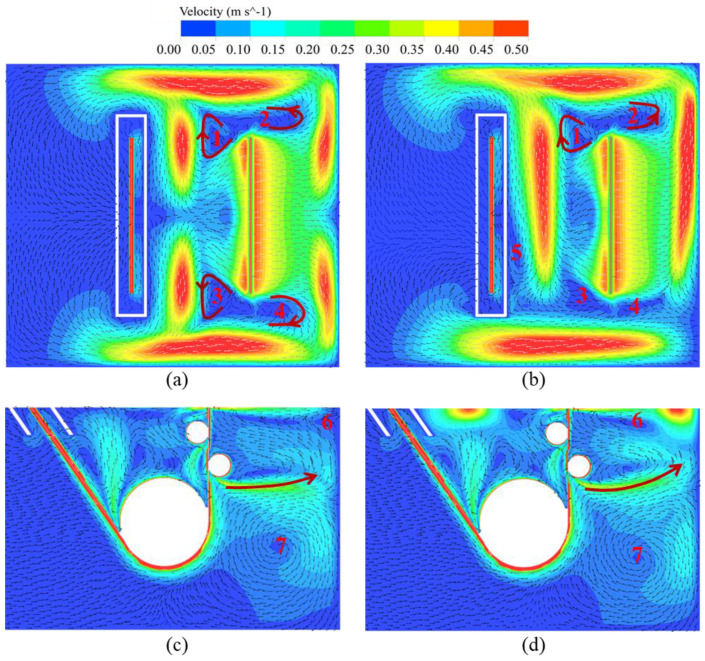
Velocity distribution on plane 1 (Z = −0.01 m) and plane 2 (Y = 0 m) with a driving current of 400 A (**a,c**) Mode 1 (**b**,**d**) Mode 2.

Due to the limited penetration depth of electromagnetic force within molten zinc, its primary impact is to improve the surface zinc flow field. Figure 12a,b presents the surface zinc velocity contours under the two EMDF modes at a driving current of 400 A. It is evident that a noteworthy alteration in the surface flow pattern has taken place, particularly with a substantial increase in flow velocity observed in the four vortex zones near the strip exit, compared to the driving current of 100 A. This variation is attributed to the electromagnetic force induced by EMDF devices on both sides of the strip, oriented along the positive *Y*-axis, which suppresses the formation of the original 3rd and 4th vortices. Regardless of the mode, the low-velocity region on the rear side of the strip exit is consistently smaller than that on the front side. For Mode 1, this phenomenon occurs due to the movement of the sink roll, which inhibits the zinc flow on the front side of the strip. For Mode 2, this occurs mainly due to the movement of molten zinc along the negative *Y*-axis, driven by the air-knife jet pressure, resulting in a lower flow velocity on the front side of the strip exit. Undoubtedly, both modes of EMDF devices significantly enhance the surface velocity of molten zinc and drive the molten zinc flow towards the rear region of the snout in a directional manner. This has a positive effect on altering the distribution of zinc dross in the vicinity of the strip exit region.

In addition, notable alterations are observed in the zinc bath internal flow field. A comparison of Figure 9c and Figure 12c reveals that the circulating flow 6 near the front end of the stabilizing roll gradually weakens. This phenomenon is primarily attributed to the excessive electromagnetic force acting on the surface zinc, which forces a substantial amount of molten zinc to rapidly flow toward the side walls of the zinc pot, thereby weakening recirculating flow 6. At the same time, the upper part of the large circulating flow 2 shows a change from a downward to an upward flow trend, replenishing the surface molten zinc. A similar pattern can be observed in Figure 9d and Figure 12d. On the other hand, it can be inferred that the upward zinc flow may facilitate the upward movement of suspended dross, allowing it to rise to the zinc bath surface and be driven away from the strip exit by EMDF.

**Figure 13:**
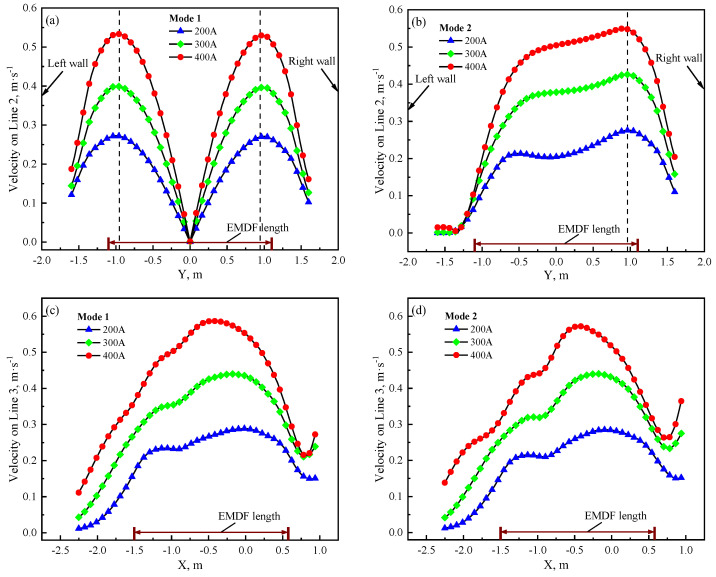
Velocity comparison with different driving currents (**a**) Mode 1 Line 2 (**b**) Mode 2 Line 2 (**c**) Mode 1 Line 3 (**d**) Mode 2 Line 3.

In this section, the velocity results on Line 2 at different driving currents are presented in Figure 13a,b. It can be observed that as the driving current increases, the maximum velocity occurs at the same position. On Line 3, the position of the maximum velocity shifts towards the negative *X*-axis direction as the driving current increases, as shown in Figure 13c,d. After reaching its peak value on Line 2, the velocity sharply decreases due to the obstruction from the zinc pot sidewalls. At this point, the upper part of Line 3, where the EMDF device is located, starts to play its role in forcing the molten zinc towards the back of the snout region. This process will ultimately determine the final distribution of surface zinc dross. With the same driving current conditions, the similarity in the maximum flow velocities observed on Line 2 and Line 3 for both modes suggests that the maximum flow velocity of the molten zinc surface relies solely on the magnitude of the electromagnetic force, irrespective of the operating length of the EMDF devices. This finding also provides valuable insights for the subsequent rational design of EMDF device parameters.

### 3.5. Comprehensive Analysis of the Surface Dross Removal for Galvanizing

**Figure 14:**
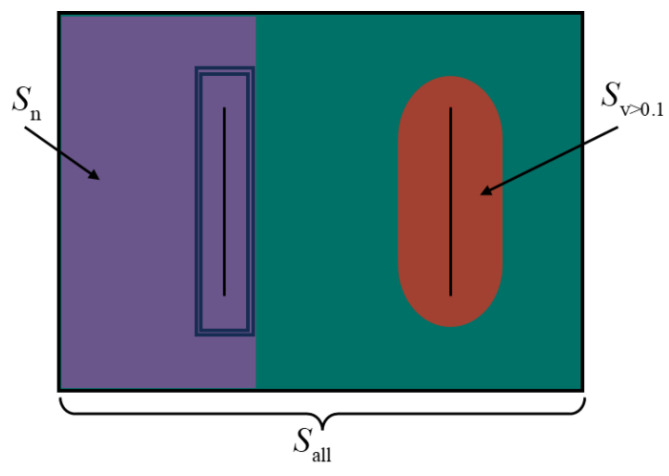
Diagrammatic representation of surface dross removal efficiency.

During the galvanizing process, the zinc dross can cause spot and stripe defects on the strip surface. To mitigate the adverse effects of zinc dross on hot-dip galvanizing production efficiency and product quality, effective control of the zinc flow is crucial. This involves optimizing the surface flow to alter the zinc dross distribution and prevent lump formation, while also considering the flow intensity and direction to avoid excessive surface flow, which may cause oxidation and overdriving. Merely studying the structure of the flow field makes it challenging to assess in detail whether the flow field facilitates the removal of zinc dross. Based on the findings from Lu et al.’s [26] investigation on the flow velocity of the zinc bath surface and the observation of the zinc dross distribution, it can be inferred that when the velocity exceeds the critical value of 0.1 m/s. In order to evaluate the dross removal quantitatively, the surface dross removal efficiency is introduced based on the surface velocity of the zinc pot, as depicted in Figure 14:

(9) $$F=\frac{S_{v\;>0.1}}{S_{\mathrm{all}}-S_{n}}$$

where, $S_{\nu >0.1}$ represents the area where the velocity exceeds 0.1 m/s on the surface molten zinc, $S_{all}$ is the total area of the surface molten zinc, and $S_{n}$ is the region of surface molten zinc behind the snout.

**Figure 15:**
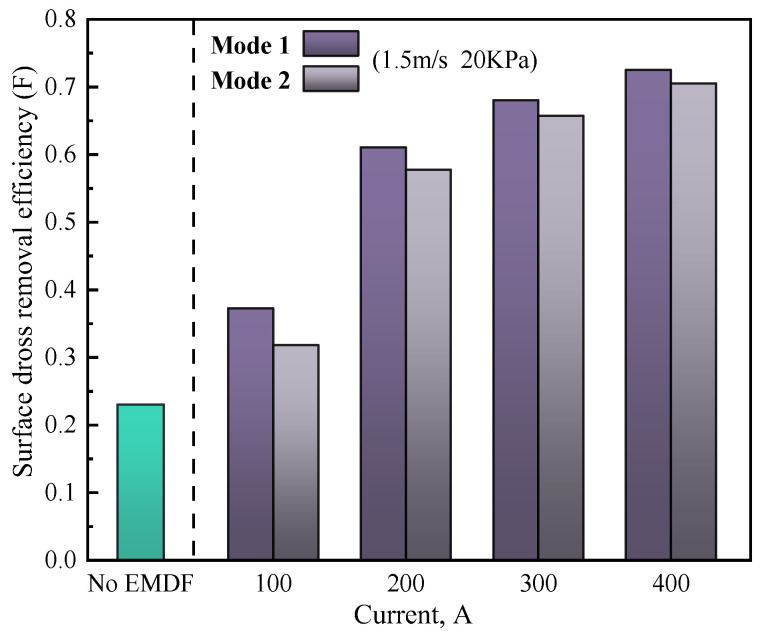
Surface dross removal efficiency comparison with and without EMDF.

The dross removal effect under different EMDF modes is shown in Figure 15, where the strip speed and air-knife jet pressure are 1.5 m/s and 20 KPa, respectively. It can be seen that when EMDF is not applied, the surface velocity in the zinc pot is so weak that the surface dross is difficult to remove to the back side of the snout, where the dross removal efficiency is only 0.25. The application of EMDF can significantly increase the removal effect of surface dross, and the F increases with the increase in the dross removal current. This is primarily due to the increased electromagnetic forces on molten zinc, which enhance its forced-flow capabilities, resulting in an increased flow velocity of the surface molten zinc. However, as the dross removal current increases from 200 A to 400 A, the increasing rate of F decreases, and the large dross removal current may cause reoxidation of the molten zinc and energy waste. Meanwhile, the dross removal effect under Mode 1 is better than that of Mode 2 for all driving currents; the larger inactive region on the free surface under Mode 2 results in a relatively poor dross removal rate.

**Figure 16:**
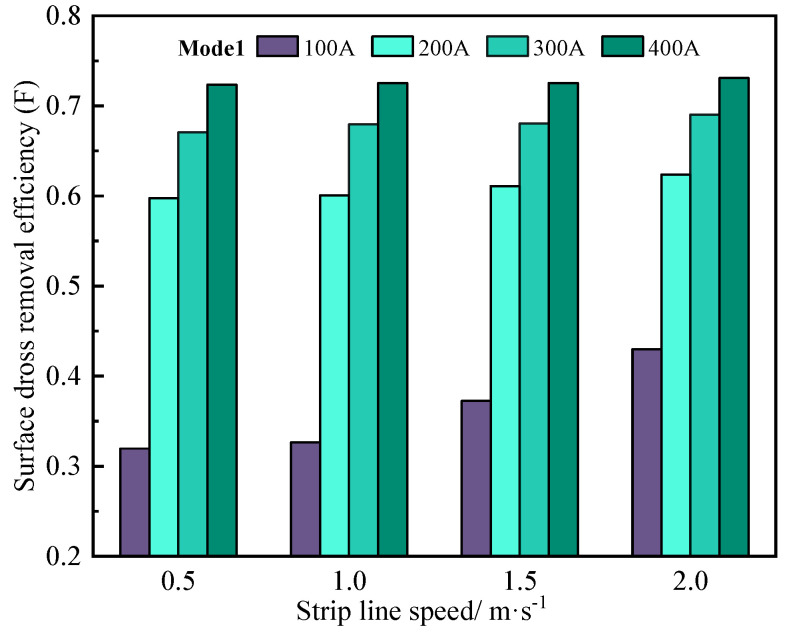
Comparison of surface dross removal efficiency at different strip speeds.

Figure 16 exhibits the surface dross efficiency under different strip line speeds and currents, where the dross removal mode of Mode 1 is applied. For the low dross removal current (100 A), F increases significantly as the strip line speed increases; the value goes up to 0.43 when the strip line speed is 2 m/s, indicating that the influence of strip line speed is predominant, while when the dross removal current is greater than 200 A, it can be seen that F is similar under different strip line speeds, indicating that the dross removal effect is mainly influenced by EMDF under the relatively high driving current.

## 4. Conclusions

In this study, a simulation investigation is presented to examine the impact of EMDF on the zinc flow field in a continuous hot-dip galvanizing line of strip steel. The influence of the air-knife jet is taken into consideration. The evaluation of the zinc bath’s capability to remove zinc dross is performed using the surface dross removal efficiency. The following conclusions are derived:

(1) Despite the limited range of air-knife jet pressure distribution near the strip steel exit, its role in facilitating the movement of the surface molten zinc should not be underestimated.
(2) The EMDF effectively optimizes the structural characteristics of the zinc bath surface flow field, forcing the zinc flow towards the side walls of the zinc pot and the rear region of the snout.
(3) The strip steel line speed and air-knife jet exhibit certain limitations in enhancing the surface dross removal efficiency. However, the EMDF substantially improves the surface dross removal efficiency, achieving a maximum value of 0.73.
(4) The depth of influence of the electromagnetic force in the zinc bath is constrained in both modes. Comparatively, the arrangement of the EMDF devices according to Mode 1 proves more advantageous in removing zinc dross in the vicinity of the strip steel exit region.

It is worth mentioning that the application of EMDF technology inevitably leads to changes in temperature and composition concentration during the flow of the zinc bath. For future research, the focus should be shifted towards enhancing the rationality of the surface dross removal efficiency by considering heat transfer and component transport aspects.

## Figures and Tables

**Table 1:** The process parameters and thermal-physical properties.

Process Parameters	Value	Process Parameters	Value
Pot size (mm × mm)	4500 × 4100 × 2020	Relative permeability	1
Strip width (mm)	2100	Electrical resistivity (Ω·m)	5.45 × 10^−8^
Strip thickness (mm)	2	Density of molten zinc (kg/m^3^)	6700
Strip line speed (m/s)	0.5, 1.0, 1.5, 2.0	Density of air (kg/m^3^)	1.225
Nozzles to strip distance (mm)	12	Viscosity of air (Pa·s)	1.7894 × 10^−5^
Nozzles to level height (mm)	300	Viscosity of molten zinc (Pa·s)	3.85 × 10^−3^
Slot jet gap (mm)	2	Current (A)	100, 200, 300, 400

## Data Availability

The original contributions presented in the study are included in the article, further inquiries can be directed to the corresponding author.
